# Septal GABA and Glutamate Neurons Express RXFP3 mRNA and Depletion of Septal RXFP3 Impaired Spatial Search Strategy and Long-Term Reference Memory in Adult Mice

**DOI:** 10.3389/fnana.2019.00030

**Published:** 2019-03-08

**Authors:** Mouna Haidar, Kimberly Tin, Cary Zhang, Mohsen Nategh, João Covita, Alexander D. Wykes, Jake Rogers, Andrew L. Gundlach

**Affiliations:** ^1^The Florey Institute of Neuroscience and Mental Health, Parkville, VIC, Australia; ^2^Florey Department of Neuroscience and Mental Health, The University of Melbourne, Parkville, VIC, Australia; ^3^Department of Anatomy and Neuroscience, The University of Melbourne, Parkville, VIC, Australia

**Keywords:** relaxin-3, RXFP3, medial septum and diagonal band of Broca, spatial and reference memory, GABA neurons, glutamate neurons, parvalbumin

## Abstract

Relaxin-3 is a highly conserved neuropeptide abundantly expressed in neurons of the *nucleus incertus (NI)*, which project to nodes of the septohippocampal system (SHS) including the medial septum/diagonal band of Broca (MS/DB) and dorsal hippocampus, as well as to limbic circuits. High densities of the G_i/o_-protein-coupled receptor for relaxin-3, known as relaxin-family peptide-3 receptor (RXFP3) are expressed throughout the SHS, further suggesting a role for relaxin-3/RXFP3 signaling in modulating learning and memory processes that occur within these networks. Therefore, this study sought to gain further anatomical and functional insights into relaxin-3/RXFP3 signaling in the mouse MS/DB. Using *Cre*/*LoxP* recombination methods, we assessed locomotion, exploratory behavior, and spatial learning and long-term reference memory in adult C57BL/6J Rxfp3^*loxP/loxP*^ mice with targeted depletion of Rxfp3 in the MS/DB. Following prior injection of an AAV^(1/2)^-Cre-IRES-eGFP vector into the MS/DB to delete/deplete Rxfp3 mRNA/RXFP3 protein, mice tested in a Morris water maze (MWM) displayed an impairment in allocentric spatial learning during acquisition, as well as an impairment in long-term reference memory on probe day. However, RXFP3-depleted and control mice displayed similar motor activity in a locomotor cell and exploratory behavior in a large open-field (LOF) test. A quantitative characterization using multiplex, fluorescent *in situ hybridization (ISH)* identified a high level of co-localization of Rxfp3 mRNA and vesicular GABA transporter (vGAT) mRNA in MS and DB neurons (~87% and ~95% co-expression, respectively). Rxfp3 mRNA was also detected, to a correspondingly lesser extent, in vesicular glutamate transporter 2 (vGlut2) mRNA-containing neurons in MS and DB (~13% and ~5% co-expression, respectively). Similarly, a qualitative assessment of the MS/DB region, identified Rxfp3 mRNA in neurons that expressed parvalbumin (PV) mRNA (reflecting hippocampally-projecting GABA neurons), whereas choline acetyltransferase mRNA-positive (acetylcholine) neurons lacked Rxfp3 mRNA. These data are consistent with a qualitative immunohistochemical analysis that revealed relaxin-3-immunoreactive nerve fibers in close apposition with PV-immunoreactive neurons in the MS/DB. Together these studies suggest relaxin-3/RXFP3 signaling in the MS/DB plays a role in modulating specific learning and long-term memory associated behaviors in adult mice via effects on GABAergic neuron populations known for their involvement in modulating hippocampal theta rhythm and associated cognitive processes.

## Introduction

The medial septum/diagonal band of Broca (MS/DB) sends strong GABAergic and cholinergic efferent projections to the hippocampal formation (Freund and Antal, 1988; Dutar et al., 1995; Vertes and Kocsis, 1997), which together with a number of other subcortical structures [e.g., supramammillary nucleus (Vertes and Kocsis, 1997) and median raphe (MR; Vertes et al., 1999)] constitute the “septohippocampal system (SHS)” (Vertes and Kocsis, 1997). This neural pathway is well-known for its important role in the control of hippocampal theta oscillations (Vertes and Kocsis, 1997; Vertes, 2005; Lubenov and Siapas, 2009), and learning and memory (Alreja et al., 2000; Wu et al., 2000), including the processing of cognitive and spatial maps (O’Keefe, 1993; Leutgeb et al., 2005; Schiller et al., 2015). Furthermore, parallel studies have also identified a population of glutamatergic neurons in the MS/DB, which can influence hippocampal theta rhythm (Colom et al., 2005; Huh et al., 2010; Robinson et al., 2016).

In addition to well-characterized forebrain subcortical regions known to modulate the SHS (for review see Vertes and Kocsis, 1997; Brown and McKenna, 2015; Korotkova et al., 2018), an important brainstem area also known for its involvement in the SHS, particularly in the rat, is the *nucleus incertus* (NI; Goto et al., 2001; Olucha-Bordonau et al., 2003; Brown and McKenna, 2015; Martínez-Bellver et al., 2015, 2017; Korotkova et al., 2018). A population of GABAergic NI neurons express the neuropeptide, relaxin-3 (Bathgate et al., 2002; Burazin et al., 2002; Ma et al., 2007), which acts via its G_i/o_-protein-coupled, relaxin-family peptide 3 receptor (RXFP3; Liu et al., 2003; Bathgate et al., 2006).

Relaxin-3 is predominantly expressed in the brain (Bathgate et al., 2002; Burazin et al., 2002; Liu et al., 2003; Sutton et al., 2004). The distribution of relaxin-3 in the brain has been studied thoroughly in the rat (Tanaka et al., 2005; Ma et al., 2007) and mouse (Smith et al., 2010). Anterograde tracing studies have revealed the NI has strong neuronal inputs to areas such as the MS/DB and hippocampus (Goto et al., 2001). In agreement with previous studies in mice (Smith et al., 2010) and rats (Ma et al., 2007), Olucha-Bordonau et al. (2012) observed dense relaxin-3 nerve fibers in the rat MS/DB, in a pattern that strongly overlaps with that of RXFP3 mRNA in the MS/DB, and other forebrain regions (Tanaka et al., 2005; Ma et al., 2007; Smith et al., 2010). NI afferent fibers were observed in close apposition to septal cholinergic and parvalbumin (PV) GABA neurons that project to the hippocampus (Olucha-Bordonau et al., 2012), suggesting the NI relaxin-3/RXFP3 system plays a functional role in modulating cognitive related behaviors via modulation of the SHS. Consistent with this hypothesis, injection of a RXFP3-selective agonist peptide [R3/I5 (Liu et al., 2005)] into the MS increased hippocampal theta rhythm in urethane-anesthetized rats, which was significantly attenuated by prior injection of a selective RXFP3 antagonist [R3(BΔ23–27)R/I5 (Kuei et al., 2007)]. In conscious rats, R3/I5 injection into the MS increased hippocampal theta rhythm in a home-cage environment, whereas injections of R3(BΔ23–27)R/I5 dose-dependently reduced hippocampal theta rhythm in rats exploring a novel, enriched context, and impaired performance in a spontaneous alternation task (SAT; Ma et al., 2009a). Collectively, these anatomical (Olucha-Bordonau et al., 2012) and functional (Ma et al., 2009a, 2013) studies on the NI relaxin-3/RXFP3 system in the rat MS/DB highlight the importance of the relaxin-3/RXFP3 system in modulating the SHS. However, similar anatomical and functional studies have not been conducted in the mouse.

Much of the research to date in mice has involved acute pharmacological treatments (Smith et al., 2014; Ma et al., 2017) which rely on the precise specificity of the peptides used for RXFP3. Additionally, other studies have used whole-of-life relaxin-3 or RXFP3 gene deletion (knockout, KO) mouse strains, which do not express the peptide or the receptor throughout development and postnatal and adult life (Sutton et al., 2009; Watanabe et al., 2011; Smith et al., 2012; Hosken et al., 2015). These KO strains may undergo neural circuit compensation or adaptation that potentially obscures key aspects of endogenous relaxin-3/RXFP3 signaling. Therefore, a global deletion of relaxin-3 signaling throughout the entire brain may conceal behavioral phenotypes that might otherwise be identified if particular brain areas are targeted for relaxin-3 or RXFP3 deletion in the mature, adult brain. Indeed, a recent study from our laboratory assessed the behavioral role of the relaxin-3/RXFP3 system in the dentate gyrus (DG) hilus of the hippocampus in floxed-Rxfp3 mice, in which conditional deletion/depletion of Rxfp3 mRNA/RXFP3 protein was achieved by local viral delivery of the Cre-recombinase restriction enzyme (Haidar et al., 2017). Depletion of RXFP3 within the DG resulted in impaired spatial reference memory in mice, in an appetitive T-maze reference memory task and sub-optimal spatial working memory in a continuous SAT in a Y-maze.

However, despite the extensive studies of the NI-SHS relaxin-3/RXFP3 system in the rat, and the latter study, the effect of relaxin-3/RXFP3 signaling in the MS/DB on spatial learning and long-term memory, and the nature of RXFP3-expressing neurons in the mouse septum has not been investigated. To address this gap in knowledge, in the present study, a similar approach was adopted and we produced a conditional deletion of Rxfp3 mRNA and depletion of RXFP3 protein in the MS/DB by local viral delivery of Cre-recombinase (Haidar et al., 2017), together with a characterization of Rxfp3 mRNA-expressing MS/DB neurons using multiplex, fluorescent *in situ hybridization (ISH)* and comparative immunohistochemistry studies.

Briefly, septal RXFP3 depletion resulted in impairment of allocentric spatial learning and impairment of long-term reference memory in the Morris water maze (MWM) paradigm. However, RXFP3-depleted and control mice displayed similar motor activity in a locomotor cell and exploratory behavior in a large open-field (LOF) test. Multiplex, fluorescent *ISH* studies demonstrated that the majority of Rxfp3 mRNA-positive neurons in the MS and DB co-express vesicular GABA transporter (vGAT) mRNA, while a correspondingly smaller population of Rxfp3 mRNA-positive neurons expressed vesicular glutamate transporter-2 (vGlut2) mRNA; and Rxfp3 mRNA was also co-expressed with PV mRNA in presumed inhibitory projection neurons. These findings are consistent with recent studies in the rat MS/DB (Albert-Gascó et al., 2018). Overall, these studies suggest ascending relaxin-3 neurons signaling via RXFP3 in the septum play an important role in modulating specific learning and long-term memory associated behaviors in adult mice, via direct effects on GABAergic neurons known for their involvement in these processes (Freund and Antal, 1988; Vinogradova, 1995; Wu et al., 2000; Tsanov, 2015; Korotkova et al., 2018).

## Materials and Methods

### Ethical Approval

All experiments were conducted with the approval of The Florey Institute of Neuroscience and Mental Health Animal Welfare Committee (AEC Application No. 13-103) and in agreement with the ethical guidelines issued by the National Health and Medical Research Council (NHMRC) of Australia. Every effort was made to ensure the mice used were treated humanely and any discomfort was kept to a minimum.

### Floxed-Rxfp3 Mice

Behavioral experiments were performed using adult, male homozygous floxed-Rxfp3 mice (C57BL/6J Rxfp3^*loxP/loxP*^) between 15 and 27 weeks of age. Male mice were used in these studies to reduce variability in anxiety-like behaviors. Inclusion of both sexes requires larger group sizes to achieve statistical power, which was not feasible. Breeding pairs of these mice were originally supplied by Janssen Pharmaceutical Companies of Johnson and Johnson (San Diego, CA, USA). These mice contain a 5′-*loxP* site located 1.4 kb upstream of the RXFP3 5′-UTR, and a 3′-*loxP* site located directly after the STOP codon and upstream of the 3′-UTR (Haidar et al., 2017). The mice were originally generated on a 129SV/B6 mixed background, but subsequently underwent multiple rounds of backcrossing onto a C57BL/6J background via a >99% purity speed congenic approach (Sutton SW, Smith CM, personal communication). Three mice died during surgery, giving a total experimental group size of 30, *n =* 12 AAV^(1/2)^-eGFP-injected control mice and *n* = 18 AAV^(1/2)^-Cre-IRES-eGFP-injected mice.

### Genotyping

In order to distinguish between wildtype (WT), heterozygous and homozygous floxed-Rxfp3 mice, mice were genotyped in-house or by Transnetyx (Cordova, TN, USA). For in-house PCR, a forward primer 5′-GGAGACAGGTCAAGAGTGATGGTCACC-3′ was used upstream of the *loxP* sequence and a reverse primer 5′-GGATAGAAGGCATGGAGTGGGAACTAGG-3′ was used downstream of the floxed region. WT mice were identified by the presence of a single 3.9 kb signal, heterozygous mice displayed a double signal of 4 kb, while homozygous mice were characterized by the amplification of a single 4.1 kb band.

### Animal Housing

Mice were group-housed (mixed treatments, ~3–4 mice per box) in open-top containers before and after surgery and during behavioral experiments. Mice were acclimatized to behavioral rooms (temperature, ~20°C; humidity, 50%) 24 h prior to testing, and were maintained on a 12-h light-dark cycle (07:00–19:00). Mice had access to standard chow and water *ad libitum*.

### Adeno-Associated Viral (AAV) Vectors

The AAV^(1/2)^-Cre-IRES-eGFP viral vector used in the present study encodes the restriction enzyme, Cre-recombinase and an enhanced green fluorescent protein (eGFP) marker, connected by an internal ribosome entry site (IRES) sequence, which allows the expression of Cre-recombinase and eGFP from a single vector (Haidar et al., 2017). The control vector, AAV^(1/2)^-eGFP (packaged in 1/2 mosaic capsid) only encoded the expression of eGFP. Transcription of both constructs was driven by the chicken beta-actin (sCAG) promoter. The final titres of the AAV^(1/2)^-Cre-IRES-eGFP and AAV^(1/2)^-eGFP vectors were ~2 × 10^11^ gc/ml.

### Stereotaxic Surgery

Mice were first placed in a small induction chamber and anesthetized with 5% isoflurane inhalation (Delvet, Seven Hills, NSW, Australia) mixed with oxygen. Once mice were anesthetized (loss of righting reflex), they were secured in a small animal stereotaxic frame (Kopf Instruments, Tujunga, CA, USA), and maintained on 1.5%–2% isoflurane (at 0.2 L/min) delivered through a mouse nasal cone. During the procedure 0.1 ml of an analgesic (Meloxicam 20 mg/kg; Troy Laboratories, Smithfield, NSW, Australia) was administered subcutaneously (Boehringer Ingelheim, St. Joseph, MO, USA) and the eyes were moistened with lubricating eye ointment (Lacri-Lube, Allergen Australia Pty Ltd, Gordon, NSW, Australia). A small midline incision was made to expose the skull and injection site. Hydrogen peroxide (6%, Gold Cross, Laverton North, VIC, Australia) was used to clean the skull and expose bregma under a dissection microscope (Leica, North Ryde, NSW, Australia).

The AAV vectors were loaded into a glass capillary injector that was connected to polyethylene tubing and a 10 μl Hamilton syringe (0.46 mm diameter, Harvard Apparatus, Holliston, MA, USA), before being mounted onto an infusion pump (PicoPlus, Harvard). A total of 1 μl was injected into the MS/DB at a rate of 0.1 μl/min. The injector was first lowered to the coordinates AP 0.85 mm, ML 0 mm and DV 4.7 mm from bregma (unless otherwise stated) based on coordinates from the mouse stereotaxic brain atlas (Franklin and Paxinos, 1997) and 0.5 μl of the virus was released. Following the first infusion, the injector was slowly raised to DV 4.3 mm, where the remaining 0.5 μl of the virus was deposited, in an attempt to ensure maximum coverage along the DV axis of the MS. After each infusion, the injector was left in place for ~5 min, before being retracted 1.0 mm and left for another min to minimize deposition of the virus along the injection tract. The skin wound was sealed with superglue and a single suture. After surgery, the mice were placed in a recovery chamber for ~1 h, where they slowly regained consciousness (30°C, Thermacage, Datesand Ltd., Manchester, UK).

### Validation of Cre-Immunoreactivity in Medial Septum/Diagonal Band of Broca (MS/DB) Neurons

In studies to confirm adequate Cre-IR in the MS before the commencement of behavioral experiments, a cohort of floxed-Rxfp3 mice (*n* = 3) was injected with AAV^(1/2)^-Cre-IRES-eGFP and transcardially perfused (4% PFA in 0.1 M PB) 21 days after stereotaxic surgery. Coronal sections (40 μm) were collected using a cryostat (Cryocut 1800, Leica Microsystems, Heerbrugg, Switzerland) into three series from ~1.94 mm to −0.10 mm from bregma at −18°C and placed in cryoprotectant solution (30% ethylene glycol, 30% glycerol, 0.05 M PB) at −20°C.

Previous studies in our laboratory have confirmed the successful deletion of the Rxfp3 gene by assessing the loss of Rxfp3 mRNA in a targeted region using *ISH* (Haidar et al., 2017). Based on these findings and the use of the same batch of AAV^(1/2)^-Cre-IRES-eGFP viral vector, it was presumed it would produce an equivalent deletion of Rxfp3 expression and depletion of RXFP3 protein in these experiments. Sections were washed 3 × 5 min with 0.1% Triton X-100 in 0.1 M PB, and then blocked in 10% NHS in 0.1% Triton X-100 and 0.1 M PB for 1 h at room temperature (RT). Sections were incubated with a polyclonal rabbit anti-Cre antibody (Novagen, Kazia Therapeutics Ltd, Sydney, NSW, Australia, cat no. 69050-3, 1:1,000) in 2% NHS and Triton X-100 in 0.1 M PB overnight at RT. The next day, the sections were washed 3 × 5 min in 0.1 M PB before being incubated in donkey anti-rabbit Alexa-594 (Invitrogen Australia, Mt Waverley, VIC, Australia, cat no. 404239, 1:500) in 0.1 M PB for 1 h at RT. Sections were then washed 3 × 5 min in 0.1 M PB and mounted onto glass microscope slides (Menzel-Glaser, Sydney, NSW, Australia). The resulting staining was observed under a Zeiss Axio Imager 2 confocal laser-scanning microscope (Carl Zeiss AG, Jena, Germany). Each section was imaged under a 10× *or* 20× objective for site validations and observation of viral spread within the MS/DB. A representative view of viral expression was generated using a 40× objective and a z-stack (1 μm intervals).

After careful observation of the viral spread and targeting within the MS/DB of both experimental groups, of the 15 mice that received an AAV^(1/2)^-Cre-IRES-eGFP viral infusion, seven were excluded from the analyses due to an insufficient level or lack of transduced neurons within the MS/DB (referred to as missed injections), and compared to the eGFP-injected group (*n* = 12). Specifically, mice were classified as missed injections if viral expression was not observed within the MS/DB or if viral expression was not observed within the bregma levels containing the MS/DB (0.26–1.18 mm). Mice included in the experimental group analysis all met the following criteria: good viral expression within the MS, with adequate spread throughout the entirety of the area, including the DB. Mice were also excluded from specific experimental test analyses if they were adjudged as outliers, defined as displaying test values that were ± two standard deviations from the group mean.

### Behavioral Testing

Three weeks after stereotaxic surgery mice were subjected to the following behavioral tests in the listed order: (1) locomotor cell test; (2) LOF test; and (3) MWM test. Mice were tested in less stressful before more stressful behavioral paradigms in an attempt to reduce any potential influence of stress on their performance. All behavioral protocols were conducted during the light phase between 09:00–19:00 h. Mice were sacrificed 9 days after the last behavioral experiment for histological analysis.

#### Locomotor Cell Test

Mice were tested in a 27 × 27 cm automated locomotor cell (Med Associates, Fairfax, VT, USA) with 70 lux light illumination, for a total duration of 1 h. Distance traveled was tracked by a photobeam array. Additionally, vertical plane entries were monitored as a measure of rearing. Data was analyzed using Activity Monitor (Version 9.02, Med Associates).

#### Exploratory Behavior: Large Open-Field (LOF) Test

In studies to assess exploratory behavior, mice were tested in a circular arena with a diameter of 1 m and their movements were tracked and analyzed using an overhead video camera and Ethovision (XT) software for a total duration of 10 min per trial. Mice were placed in the center region of the arena upon initiation of the trial. Measures used to reflect exploratory behavior included the total distance traveled during the trial and the number of times mice made entries into the defined outer and center circles (Nielson et al., 1965).

#### Morris Water Maze (MWM) Test

In tests to measure spatial learning and spatial long-term reference memory, mice were trained in a circular pool (1.2 m diameter) to locate a hidden platform (10 cm diameter), which was submerged ~0.5–1 cm under the surface of the water, which was made opaque using non-toxic white paint. The temperature of the pool was monitored regularly to ensure it was 18–24°C at all times. The pool was surrounded by various 2D and 3D cues designed to assist the mice to find the platform (Rogers et al., 2017). The acquisition phase of the test involved six training days, with four trials per day for each mouse. For each trial, the mice were put facing the pool wall in a semi-random starting position using the compass points (N, NW, E and SE). They were allowed to swim for a total duration of 2 min or until they located the hidden platform. If a mouse failed to locate the platform within the allocated time, it was guided to the platform. The mouse then had to remain on the platform for a further 30 s, before being removed from the pool and returned to its home cage and dried under a heat lamp. For the acquisition sessions, the mean latency was calculated by averaging the latency obtained over the four trials. Approximately 24 h after the last training day, on probe day, the platform was removed from the target quadrant (SW) and the mice were allowed to swim freely for 2 min. The percent time spent in the target quadrant compared to the adjacent and opposite quadrants, as well as the number of platform crossings, was calculated.

### Statistical Analysis

All graphical and statistical analysis was done using Graphpad Prism 6 for Windows (Graphpad, La Jolla, CA, USA). Once the data from mice with missed Cre injections were excluded from analysis, behavioral data and data sets that met the assumption of normality were analyzed using either a one-way analysis of variance (ANOVA), two-way repeated measures ANOVA or an unpaired *t*-test. Bonferroni's test for *post-hoc* corrections was used. Results were considered significant if the *p*-value was less than 0.05. For MWM search strategy analysis, Stata (Survey Design and Analysis Services Pty Ltd, ACT, Australia) was used to statistically discriminate between non-spatial (egocentric) or spatial (allocentric) learning during training days, as described (Rogers et al., 2017). The % spatial search strategy was used as an indicator of allocentric spatial learning.

### Multiplex *in situ* Hybridization (ISH)

The distribution of MS/DB Rxfp3 mRNA-positive neurons and their neurochemical phenotype was assessed using multiplex *ISH* (RNAscope™, Advanced Cell Diagnostics, Hayward, CA, USA) as described (Albert-Gascó et al., 2018). Briefly, 3 naïve mice were deeply anesthetized with 5% isoflurane, and brains were quickly removed, rapidly frozen on dry ice and embedded in O.C.T. (Tissue-Tek, Sakura Finetek, Torrance, CA, USA). Coronal sections (16 μm) from the mid, anterior part of the MS/DB (0.74 mm from bregma) were cut and slide-mounted on Superfrost-Plus slides (Fisher Scientific, Hampton, NH, USA). Sections were fixed (4% PFA in 0.1 M PB) for 16 min at RT and processed according to the manufacturer’s protocol (Advanced Cell Diagnostics) using combinations of specific probes for Rxfp3, vGAT, vGlut2, PV and ChAT mRNA. Following ISH, sections were stained with DAPI, coverslipped with Fluoromount-G (Southern Biotech, Birmingham, AL, USA) and imaged.

In order to differentiate between MS and DB (composed of vertical DB and horizontal DB) subregions for quantification, the different area borders in the corresponding brain atlas sections were superimposed onto the maximum intensity projection obtained from the stacked images. Using ImageJ (Version 1.52g, NIH, Bethesda, MD, USA), automated cell counting was applied for cell nuclei of the delineated areas. Those species of mRNA (Rxfp3, vGAT and vGlut2) quantitatively assessed in labeled neurons were counted manually using ZEN Blue Lite software (Carl Zeiss, Jena, Germany) and the degree of co-localization of Rxfp3 with vGAT and vGlut2 mRNA was determined using two coronal sections from the mid, anterior part of the MS/DB (0.74 mm from bregma) from three mice.

### Immunohistochemistry for Relaxin-3, Parvalbumin (PV) and Choline Acetyltransferase (ChAT)

Mice were transcardially perfused (4% PFA in 0.1 M PB) and brains were dissected and post-fixed in 4% PFA in 0.1 M PB for 1 h at 4°C before transfer to 20% sucrose in 0.1 M PB and storage at 4°C overnight. The next day, brains were embedded in O.C.T. (Tissue-Tek), frozen over dry ice, wrapped in Parafilm (Bemis, Oshkosh, WI, USA) and stored at −80°C.

In studies to detect the relationship between relaxin-3-positive nerve fibers and other markers of MS/DB neurons, a double-label immunohistochemistry protocol was used. Ten (10) week old male mice (*n* = 3) were injected with colchicine (20 μg in 5 μl) into the lateral ventricle at 1 μl per min, using the stereotaxic procedure described. Mice were transcardially perfused (4% PFA in 0.1 M PB) ~16–24 h post-surgery and post-fixed in 4% PFA in 0.1 M PB for 1 h at RT, before transfer to a 20% sucrose solution in 0.1 M PB and storage overnight at 4°C. The following day, the brains were embedded in O.C.T. (Tissue-Tek) and frozen over dry ice, before being stored at −80°C until use. Coronal sections (40 μm) were collected into four series spanning from bregma levels −1.22 mm to −3.80 mm using a cryostat at −18°C and stored in cryoprotectant solution (30% ethylene glycol, 30% glycerol, 0.05 M PB) at −20°C.

Sections were washed for 3 × 5 min each with 0.1% Triton X-100 in 0.1 M PB, and then blocked in 10% NHS in 0.1% Triton X-100 and 0.1% PB for 1 h at RT. Tissue were then incubated with: (1) a monoclonal mouse anti-relaxin-3 antibody (HK4-144-10; Kizawa et al., 2003; Tanaka et al., 2005; Ma et al., 2013; 1:5 dilution); (2) a polyclonal goat anti-ChAT antibody (Merck Millipore, Bayswater VIC, Australia, cat no. AB144P, 1:500 dilution); and (3) a polyclonal rabbit anti-PV antibody (Abcam, Cambridge, UK, cat no. ab11427, 1:1,000 dilution), in 2% NHS and 0.1% Triton X-100 in 0.1 M PB overnight at RT. The following day, the sections were washed for 3 × 5 min in 0.1 M PB before incubation in secondary antibody. The secondary antibodies used for incubation included: (1) donkey anti-mouse Alexa 488 (Jackson Immunoresearch, West Grove, PA, USA, cat no. 715545150, 1:500 dilution; (2) donkey anti-rabbit Alexa 647 (JIR, 711605152, 1:500 dilution); and (3) donkey anti-goat Alexa 594 (Jackson Immunoresearch, cat no. 705585147, 1:500 dilution). They were incubated in 0.1 M PB for 1 h at RT. Following the secondary antibody incubation, sections were washed for 3 × 5 min in 0.1 M PB, before being mounted onto glass slides (Menzel-Glaser).

### Microscopy

Resultant staining was observed under a LSM 780 Zeiss Axio Imager 2 confocal laser-scanning microscope (Carl Zeiss AG, Jena, Germany). Putative contacts between relaxin-3-positive nerve fibers and PV-, CR- and ChAT-positive neurons were examined with a 20× objective, and further investigations were conducted using a 63× objective. Images at a single z-plane or as a z-stack at 0.1 μm intervals were collected, as described (Haidar et al., 2017). For mRNA quantification, a mosaic z stack of the entire MS and DB was imaged from slides labeled for Rxfp3, vGAT and vGlut2 mRNA using a 40× objective at 0.8 μm intervals. Higher resolution representative z-stack images (insets) from a single x and y position were imaged using a 63× objective at 0.3 μm intervals.

## Results

### Confirmation of AAV^(1/2)^-Cre-IRES-eGFP Expression in MS/DB Neurons

**Figure 1:**
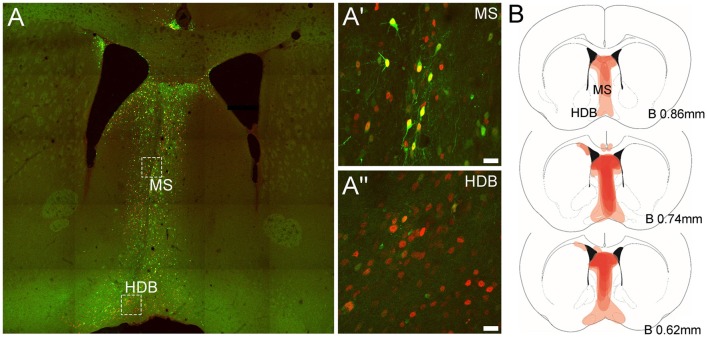
Representative expression of green fluorescent protein (eGFP) and Cre-recombinase and viral spread in medial septum/diagonal band of Broca (MS/DB) in mice injected with the AAV^(1/2)^-Cre-IRES-eGFP viral vector. **(A)** Tiled (20× mosaic) image illustrating the presence of eGFP (green) and Cre-recombinase (red) in neurons in the MS/DB. **(A′,A″)** High magnification images (63× maximum projections) of boxed areas in **(A)** in MS and HDB, revealing the presence of endogenous eGFP and Cre-recombinase IR in both regions, with some neurons (yellow) strongly expressing both proteins and other neuronal cell-bodies expressing stronger Cre-IR than eGFP, consistent with the design of the viral vector used.** (B)** Schematic images documenting the relative level of Cre-recombinase expression (light/dark red shading) observed at different bregma levels containing the MS, HDB and VDB in the C57BL/6J floxed-Rxfp3 mice (*n* = 8) analyzed in the behavioral studies. Darker areas represent those sites where the viral-driven Cre-recombinase expression was consistently concentrated in most or all mice at that level.

In order to confirm that the AAV^(1/2)^-Cre-IRES-eGFP vector drove Cre-recombinase expression *in vivo* and transduced MS and DB neurons, sections from adult floxed-Rxfp3 mice injected in the MS with AAV^(1/2)^-Cre-IRES-eGFP were processed for Cre-immunoreactivity (IR) and eGFP fluorescence. Examination of these sections revealed a substantial number of MS and DB neurons expressing eGFP- and Cre-IR (Figures 1A–A″). Importantly, AAV^(1/2)^-Cre-IRES-eGFP viral transduction was observed in areas of the MS and DB that contain high levels of Rxfp3 mRNA (Ma et al., 2007; Smith et al., 2010), with viral spread along the rostrocaudal extent of the MS/DB region spanning +1.18–0.38 mm relative to bregma (see viral spread in a mouse representative of those included in the behavioral analysis; Figure 1B).

### RXFP3 Depletion From MS/DB

#### Impaired Spatial Search Strategy Selection and Long-Term Reference Memory in a Morris Water Maze

**Figure 2:**
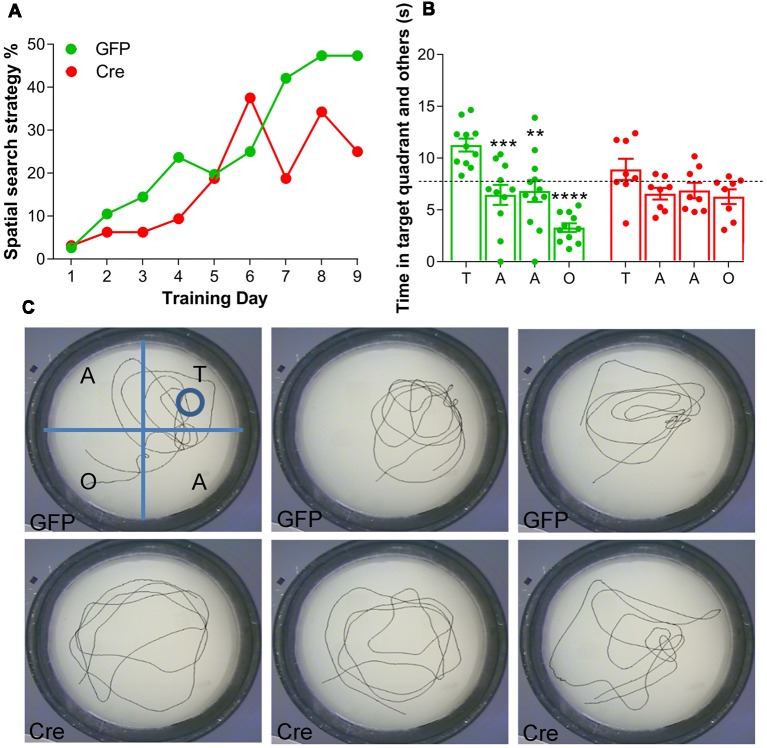
Effect of RXFP3 depletion in the MS/DB of adult, floxed-Rxfp3 mice on learning and long-term reference memory in the Morris Water Maze (MWM) test. **(A)** Mice with RXFP3 depleted from the MS/DB were less likely to adopt a spatial search strategy across the acquisition days, relative to eGFP-injected control mice [treatment: OR: 0.56, 95% CI (0.33; 0.96), *p* = 0.034]. **(B)** On the probe day, AAV^(1/2)^-eGFP mice spent significantly more time in the target quadrant relative to the other quadrants in the first 30 s bin, whereas, AAV^(1/2)^-Cre-IRES-eGFP mice did not spend more time in the target quadrant relative to the other quadrants in the same 30 s period (one-way analysis of variance (ANOVA), Bonferroni *post-hoc* analysis, **p < 0.01, ***p < 0.001, ****p < 0.0001, *n* = 8–12 mice per group).** (C)** Examples of swim tracks during the first 30 s on probe day of three representative (upper) AAV-eGFP injected mice (GFP1–3) and (lower) AAV^(1/2)^-Cre-IRES-eGFP mice (Cre1–3).

The first objective of this test was to assess search strategy selection during acquisition in a MWM. An analysis of search strategies indicated that both AAV^(1/2)^-Cre-IRES-eGFP and AAV^(1/2)^-eGFP injected mice adopted spatial learning over acquisition days, as the adjusted odds ratio (OR) of adopting an allocentric search strategy increased by ~30% per acquisition day (learning effect, day: OR: 1.28, 95% CI (1.22; 1.35), *p* < 0.001, *n* = 8–11 per group, Figure 2A). Importantly, a significant treatment effect was observed, whereby AAV^(1/2)^-Cre-IRES-eGFP mice were 44% less likely to adopt an allocentric search strategy, relative to AAV^(1/2)^-eGFP control mice (treatment: OR: 0.56, 95% CI (0.33; 0.96), *p* = 0.034, Figure 2A), suggesting an impairment in mice adopting an allocentric search strategy during acquisition, following RXFP3 depletion in the MS/DB, relative to control mice.

Twenty-four hours after the last acquisition day, a probe trial was conducted. AAV^(1/2)^-eGFP mice spent significantly more time in the target quadrant relative to the other quadrants in the first 30-s time bin (1-way ANOVA, *F*_(7,69)_ = 8.60, *p* < 0.0001, Figure 2B). A Bonferroni’s planned comparisons test revealed AAV^(1/2)^-eGFP control mice spent significantly more time in the target quadrant than in the adjacent quadrant to the right (*p* < 0.001), the adjacent quadrant to the left (*p* < 0.005) and to the opposite quadrant (*p* < 0.0001), suggesting intact long-term reference memory. Conversely, AAV^(1/2)^-Cre-IRES-eGFP mice did not spend more time in the target quadrant relative to the other quadrants in a 30 s time bin (Bonferroni planned comparisons, all *p* > 0.05), suggesting depletion of RXFP3 from MS/DB results in an impairment in long-term reference memory on probe day in a MWM, relative to performance of control, eGFP-injected mice.

The time spent in the target quadrant was compared with chance values of 7.5 s and 30-s for the 30 s time bin. The chance value is the amount of time a mouse would be expected to spend in the target quadrant if no spatial memory had been formed (Porte et al., 2008). AAV^(1/2)^-eGFP control mice spent significantly greater than 25% of their time in the target quadrant in the 30 s time bin (*t*_(20)_ = 6.00, *p* < 0.0001), whereas AAV^(1/2)^-Cre-IRES-eGFP mice performed at chance during the 30-s time bin (*t*_(14)_ = 1.37, *p* = 0.19). Probe day swim patterns from three representative mice from each treatment group (Figure 2C), demonstrate that AAV^(1/2)^-Cre-IRES-eGFP mice often displayed a “random” or a “scanning” search strategy on probe day, whereas AAV^(1/2)^-eGFP mice were more likely to utilize “chaining” or a “directed” search strategy, and display a preference for the target area.

#### No Effect on General Locomotor Activity or Exploratory Behavior in the Large Open-Field Test

Both treatment groups displayed a similar distance traveled during a 1 h locomotor activity test (*t*_(18)_ = 1.023, *p* = 0.320, Table 1). This result was consistent across 5-min time bins (RM 2-way ANOVA, main effect of treatment, *F*_(2,24)_ = 0.917, *p* = 0.413, Table 1), and a significant reduction in distance traveled over time was observed in both treatment groups (main effect of time, *F*_(11,264)_ = 5.888, *p* < 0.0001; treatment × time interaction, *F*_(22,264)_ = 0.665, *p* = 0.872). RXFP3 depletion in the MS/DB did not induce any changes, relative to AAV^(1/2)^-eGFP control mice, in the total distance traveled (*t*_(18)_ = 0.6906, *p* = 0.486, Table 1), or the total time spent in the center (*t*_(18)_ = 0.3803, *p* = 0.7082).

**Table 1:** General locomotor activity and exploratory behavior of AAV^(1/2)^-eGFP and AAV^(1/2)^-Cre-IRES-eGFP treated mice.

Behavioral test	Measure	GFP	Cre
Locomotor activity	Total distance traveled (m)	136 ± 18.2	113 ± 7.33
Exploratory behavior	Total distance traveled (cm)	853 ± 40.2	890 ± 24.3
Large open field test	Time spent in center (s)	30.7 ± 2.17	29.3 ± 3.44

*Data are expressed as mean ± SEM, *n* = 8–12 mice per group*.

### Rxfp3 mRNA-Positive Neurons in MS/DB Express vGAT or vGlut2 mRNA

**Figure 3:**
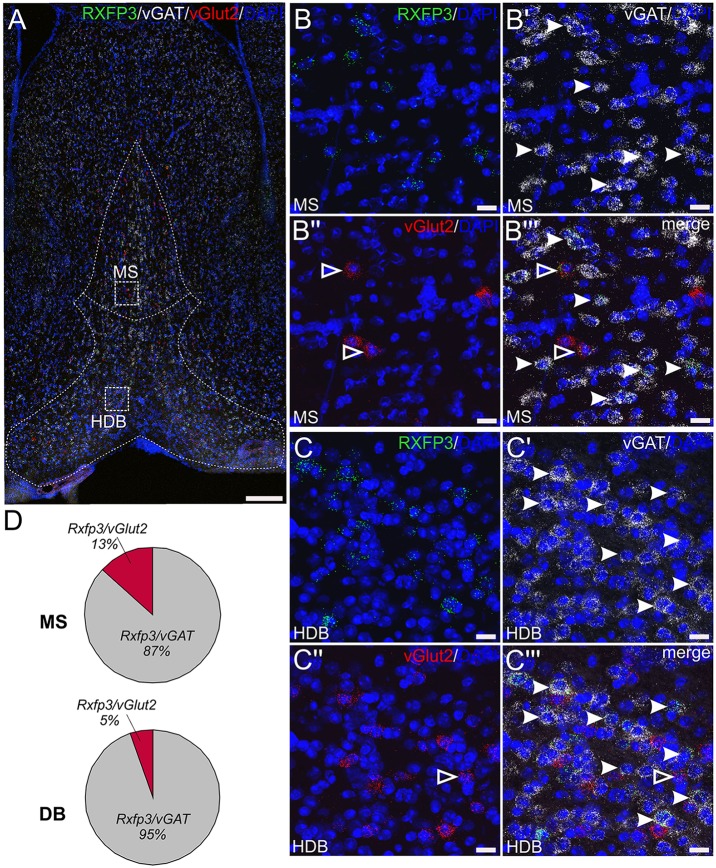
Detection of Rxfp3 mRNA and co-expression with vGAT and vGlut2 mRNA in the MS/DB of adult mice. **(A)** Distribution of neurons expressing Rxfp3 (green), vGAT (white) and vGlut2 (red) mRNA at 0.74 mm from bregma (40× images represented as a maximum projection, 0.8 μm intervals). Dotted lines were drawn and superimposed on the maximum intensity projection from the corresponding mouse brain atlas section. Sections were counterstained with DAPI (blue) to indicate cell nuclei. **(B,C)** High magnification images (40× images represented as maximum projection, 0.3 μm intervals) of the boxed areas in **(A)** illustrating the co-localization of Rxfp3 **(B,C)**, vGAT **(B′,C′)**, vGlut2 **(B″,C″)** mRNA and the merged channels **(B″′,C″′)** in the MS and horizontal DB. Examples of neurons co-expressing Rxfp3 and vGAT mRNA (solid arrowheads) and Rxfp3 and vGlut2 mRNA (open arrowheads) are indicated. **(D)** Percentage of co-localization of Rxfp3 mRNA and vGAT and vGlut2 mRNA, respectively within the MS and DB. Scale bars 200 μm **(A)** and 20 μm **(B,C)**.

In studies to assess the phenotype of RXFP3-expressing neurons in mouse septum, multiplex *ISH* was used to investigate the relative co-expression of Rxfp3 mRNA with vGAT and vGlut2 mRNA, in the mid, anterior part of the MS/DB (0.74 mm from bregma; Table 2; Figure 3). The pattern of septal Rxfp3 mRNA-containing neurons identified is in agreement with that observed in previous studies in mouse, and the pattern of relaxin-3-positive nerve fibers/terminals in mouse (Smith et al., 2010). Rxfp3 mRNA-expressing neurons were detected predominantly in the more lateral region of the MS, bordering the lateral septum (LS), and in the vertical and horizontal regions of DB (Figure 3A). The majority of Rxfp3 mRNA-positive neurons in the MS co-expressed vGAT mRNA (65/74 neurons, ~87%), while only 10/74 (~13%) Rxfp3 mRNA-positive neurons co-expressed vGlut2 mRNA (Figures 3B–B″′,D; Table 2). Similarly, within the vertical and horizontal DB, the majority of Rxfp3 mRNA-positive neurons co-expressed vGAT mRNA (119/127 neurons, 95%), while only 7/127 (~5%) Rxfp3 mRNA-positive neurons expressed vGlut2 mRNA (Figures 3C–C″′,D; Table 2). Collectively, the GABAergic phenotype of MS/DB Rxfp3 mRNA-expressing neurons identified in the present study is in agreement with recent studies in the rat septal region (Albert-Gascó et al., 2018).

**Table 2:** Number of neurons expressing relaxin-family peptide-3 (Rxfp3), vesicular GABA transporter (vGAT) and vesicular glutamate transporter-2 (vGlut2) mRNA and relative co-expression in the mid anterior MS/DB on the mouse.

Area	DAPI	Rxfp3	vGAT	vGlut2	Rxfp3/vGAT	Rxfp3/vGlut2
MS	890 ± 75	74 ± 5	394 ± 22	75 ± 5	65 ± 5	10 ± 1
DB	1929 ± 38	127 ± 14	787 ± 51	122 ± 28	119 ± 12	7 ± 2

*Data are expressed as mean ± SEM. Neuron counts and the degree of co-localization of Rxfp3 mRNA with vGAT and vGlut2 mRNA were determined using two coronal sections from the mid, anterior part of the MS/DB (0.74 mm from bregma) from *n* = 3 mice. DAPI staining was used to calculate the total number of neurons within the anatomical area assessed. MS, medial septum; DB, diagonal band (horizontal and vertical regions)*.

### Rxfp3 mRNA-Positive Neurons in MS/DB Express PV, but Not ChAT mRNA

**Figure 4:**
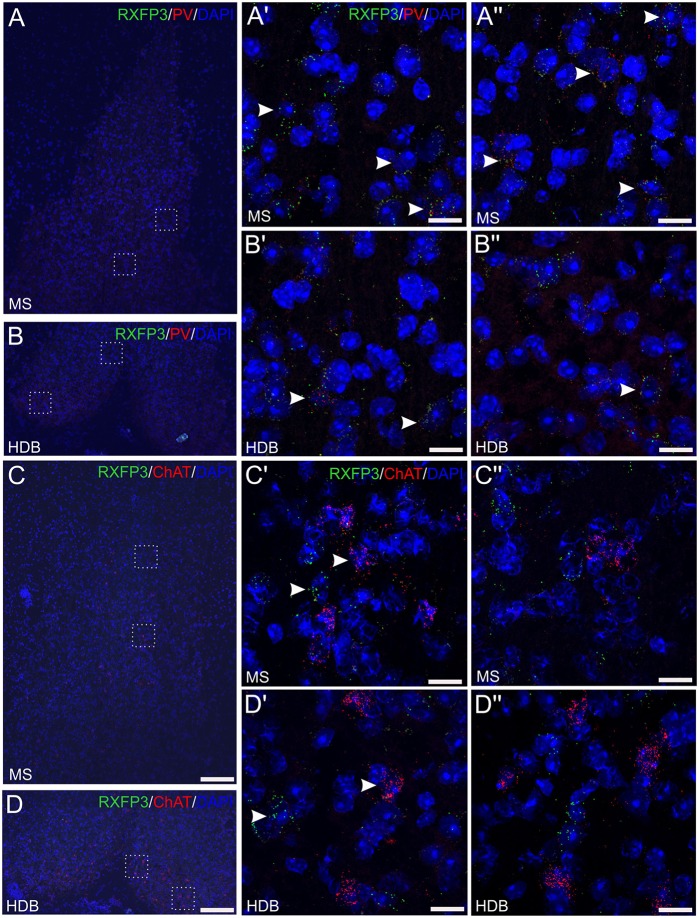
Detection of Rxfp3 mRNA and co-expression with parvalbumin (PV) mRNA, but not ChAT mRNA, in the MS/DB of adult mice. **(A,B)** Detection of Rxfp3 and PV mRNA in the MS **(A,A″)** and horizontal DB **(B,B″)**. Low-magnification images of the MS/DB area (left) and high-magnification images (63× images represented as maximum projections, 0.3 μm intervals) of the boxed areas in (**A,B**; right), illustrate the presence of Rxfp3 (green) and PV (red) mRNA, in both distinct neurons and the same neurons, consistent with RXFP3 expression by a population of PV-positive GABA neurons. Sections were counterstained with DAPI (blue) to indicate cell nuclei. Arrowheads indicate Rxfp3 mRNA in PV mRNA-positive neurons in the different fields. **(C,D)** Detection of Rxfp3 and ChAT mRNA in the MS **(C,C″)** and horizontal DB **(D,D″)**. Low-magnification overview images (left) and high-magnification images of the boxed areas in (**C**,**D**; right) illustrate the presence of strong Rxfp3 (green) and ChAT mRNA (red) expression in multiple neurons (arrowheads), but provide no evidence of co-expression of Rxfp3 (green) and ChAT mRNA in either MS or DB. Sections were counterstained with DAPI to indicate cell nuclei (blue). Scale bars 200 μm **(A–D)** and 20 μm **(A′–D″)**.

In the material examined, Rxfp3 and PV mRNA were observed in the same neurons in all regions (Figures 4A–B″), with consistent co-expression in the horizontal DB, while there was no evidence of Rxfp3 and ChAT mRNA co-expression in MS or regions of the DB (Figures 4C–D″). Using this combination of mRNA probes, there were neurons that expressed *only* Rxfp3 mRNA (Figures 4A′–D″), consistent with the presence of populations of MS/DB inhibitory or excitatory neurons that are responsive to relaxin-3 signaling, but do not express PV (or ChAT). This expression pattern is consistent with the high level of co-expression of Rxfp3 mRNA with vGAT mRNA observed in the present study (Figure 3) and in the rat septum (Albert-Gascó et al., 2018).

### Relaxin-3-Positive Nerve Fibers in Close Apposition With PV- (and ChAT)-Positive Neurons in MS/DB

**Figure 5:**
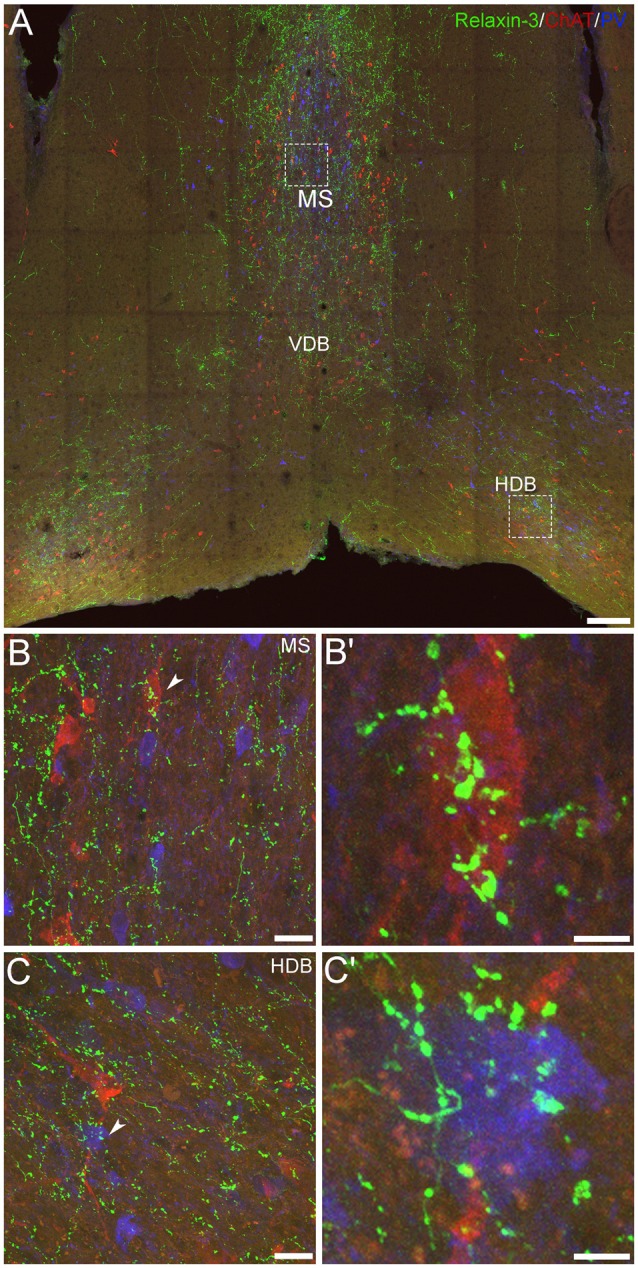
Comparative distribution of relaxin-3-immunoreactive nerve fibers and neurons positive for GABAergic and cholinergic markers in the MS/DB of adult mice. **(A)** A mosaic digital image (20× images represented as z-stack maximum projections, 1 μm intervals) of mouse MS/DB at 0.74 mm from bregma, illustrating relaxin-3 (green), ChAT (red) and PV (blue) immunoreactivity. **(B,C)** High-resolution images (63×, z-stack maximum projections, 0.1 μm intervals) of the boxed areas in **(A)**, illustrate the potential close association of relaxin-3 elements with a ChAT-immunoreactive neuron in MS **(B,B′)** and a PV-immunoreactive neuron in horizontal DB **(C,C′)**. Scale bars 200 μm **(A)**, 20 μm **(B,C)** and 5 μm **(B′,C′)**.

In previous immunohistochemical studies, neuronal fibers containing relaxin-3-IR were observed throughout the entire rostrocaudal extent of the mouse MS/DB, in a pattern that strongly overlapped the distribution of Rxfp3 mRNA observed in mouse (Smith et al., 2010) and rat brain (Ma et al., 2009a; Olucha-Bordonau et al., 2012). Therefore, in light of earlier studies in rat MS/DB, reporting putative synaptic contacts between NI projections (and relaxin-3-positive elements) with both PV- and ChAT-positive neurons in MS/DB (Olucha-Bordonau et al., 2012), a triple-label immunohistochemistry protocol was employed in an effort to analyze putative contacts between relaxin-3 nerve fibers/terminals and PV- and/or ChAT-positive neuronal soma and processes in mouse MS/DB. After obtaining 20× z-stack tile scans at 1 μm z-intervals of the overall regions of interest, and representative 63× z-stacks at 0.1 μm z-intervals of the MS and DB, a qualitative analysis of the interactions between the fibers and neurons was conducted. Neuronal fibers containing relaxin-3-IR were observed near adjacent to some ChAT-immunoreactive neurons and PV-immunoreactive neurons in the MS (Figures 5A–B′). Similarly, relaxin-3-positive nerve fibers were observed adjacent to ChAT- and PV-positive neurons in the DB (Figures 5C,C′), with a higher proportion of PV-positive neurons receiving a putative innervation in this region than in MS (see Olucha-Bordonau et al., 2012).

However, while these findings suggest a direct synaptic regulation by relaxin-3 of these septal neuron populations, parallel evidence of the phenotype of RXFP3-expressing neurons in MS/DB obtained using multiplex *ISH* (Figures 3, 4) suggest that relaxin-3/RXFP3 signaling does not directly impact ChAT mRNA-positive cholinergic neurons in the mouse or the rat MS/DB (Albert-Gascó et al., 2018), and more precise identification of the structural nature of relaxin-3/RXFP3 actions on PV neurons will require labeling with synaptic marker proteins and/or higher resolution light or electron microscopic studies.

## Discussion

In the present study, in light of the putative role of the relaxin-3/RXFP3 signaling system within the SHS, we assessed the behavior of mice after specific depletion of RXFP3 within the MS/DB; and conducted a histological analysis employing immunohistochemistry and multiplex *ISH* to investigate the possible association of relaxin-3-positive nerve fibers with GABAergic and/or cholinergic neurons, and the GABAergic or glutamatergic phenotype of Rxfp3 mRNA-positive neurons, respectively, within the mouse septal region.

In this initial study to manipulate relaxin-3/RXFP3 signaling within the mouse septum using a recently validated viral method for the specific Cre-recombinase-induced depletion of RXFP3 from the MS/DB of *floxed*-Rxfp3 (C57BL/6J Rxfp3^*loxP/loxP*^) mice (Haidar et al., 2017), we observed impairment in allocentric spatial learning during acquisition and impairment in long-term reference memory on probe day, in the MWM test. In contrast, the depletion of septal RXFP3 did not affect general locomotor activity and exploratory behavior in a locomotor cell and LOF test, respectively, suggesting the effect on optimal memory processes is quite selective. Notably, the alterations in learning and memory observed in correctly targeted Cre-treated mice were observed relative to the behavior of mice injected with an identical virus expressing a control (Cre-negative) vector, consistent with the effects observed being due to RXFP3 depletion. Indeed, in an earlier study employing an identical protocol, we directly assessed Cre-dependent Rxfp3 mRNA depletion in the target area of hippocampus (Haidar et al., 2017); but unfortunately, attempts to obtain similar data in the current study using 40 μm PFA-fixed sections from mice tested behaviorally, and fluorescent *ISH* detection of Rxfp3 mRNA, were unsuccessful (data not shown).

In a quantitative characterization using multiplex, fluorescent *ISH*, we identified a high level of co-localization of Rxfp3 mRNA and vGAT mRNA in MS and DB neurons (~87% and ~95% co-expression, respectively). Rxfp3 mRNA was also detected to a correspondingly lesser extent in vGlut2 mRNA-containing neurons in MS and DB (~13% and ~5% co-expression, respectively). Similarly, a qualitative assessment of the MS/DB region, identified Rxfp3 mRNA in neurons that expressed PV mRNA, consistent with RXFP3 expression by a population of septal GABAergic neurons that project to the hippocampus (Unal et al., 2015; Gangadharan et al., 2016), whereas ChAT mRNA-positive (cholinergic) neurons lacked Rxfp3 mRNA expression, consistent with recent studies in the rat (Albert-Gascó et al., 2018). Interestingly, our qualitative immunohistochemical studies revealed relaxin-3-immunoreactive nerve fibers in close apposition with PV-immunoreactive neurons (and ChAT-immunoreactive neurons) in the MS/DB. These data are consistent with results using a similar approach in hippocampus of mouse (Haidar et al., 2017) and rat (Rytova et al., in press), where relaxin-3 neurons were observed in close contact with SST/GABA neurons; and with reports of labeled boutons derived from NI projections making close contacts on ChAT-immunoreactive neurons in rat MS/DB (Olucha-Bordonau et al., 2012). However, given the contrast of the current immunohistochemical data with the pattern of Rxfp3 mRNA localization in the MS/DB, the findings must be interpreted cautiously, as the existence (or absence) of relaxin-3-containing synapses on PV- (or ChAT-) immunoreactive neurons was not verified using synaptic markers and high resolution imaging.

Overall, this study identified key behavioral consequences of presumed RXFP3 depletion from the mouse MS/DB, providing timely new insights into the complex modulation of learning and memory processes by this neuropeptide system within the SHS. Indeed, the MS and hippocampus have been studied extensively, particularly in past decades, in relation to learning and memory, particularly using electrical and excitotoxic lesions or broad inhibition with GABA agonists, such as muscimol, or acute excitation with glutamate or NMDA (Decker et al., 1995; Leutgeb and Mizumori, 1999; Krebs and Parent, 2005). More recently, however, there has been more focused studies on the role of specific septal neuronal types in the regulation of learning and memory (Alreja et al., 2000; Wu et al., 2000) and associated neurophysiological activity of the hippocampus, particularly theta rhythm (Robinson et al., 2016). Indeed, in the mouse, the GABAergic innervation from the MS and DB complex contributes to temporal coordination of neuronal activity via several types of cortical GABAergic interneurons in both hippocampus and extra-hippocampal cortices. Oscillatory septal neuronal firing at delta, theta, and gamma frequencies may phase interneuron activity (Unal et al., 2015).

**Figure 6:**
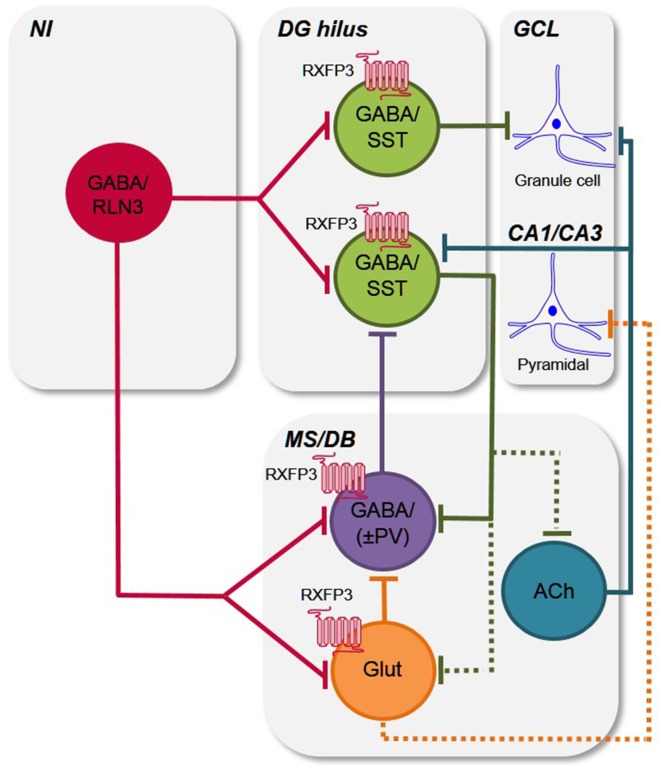
Putative circuits by which relaxin-3/RXFP3 signaling modulates activity in the hippocampus and medial septum/diagonal band of Broca. Simple schematic circuit diagram to illustrate how the relaxin-3/RXFP3 system is anatomically positioned to modulate the activity of the septohippocampal and hippocamposeptal systems, which are important for cognitive functions and related behaviors (Tsanov, 2015; Korotkova et al., 2018; Müller and Remy, 2018). Based on previous and current findings (see “Discussion” section), the ascending relaxin-3 projections from the NI innervate local and/or projection GABA/SST neurons in the mouse DG hilus (Haidar et al., 2017), and the GABA/PV+ve hippocampal projecting neurons and possibly other GABA interneurons (i.e., GABA/(PV−ve), as well as some glutamate neurons in the MS/DB, and modulate their activity via RXFP3 (current study; see e.g., Müller and Remy, 2018 for recent review). Existing data also indicates that neurons present in the other nodes of the septohippocampal system (SHS) are RXFP3-positive, but as their neurochemical identity is currently unknown, they are omitted for clarity. Furthermore, any possible collateralization of relaxin-3 neurons and *all* possible intra-MS/DB interconnections are not illustrated for clarity. Abbreviations: ACh, acetylcholine; CA1/CA3, CA1 and CA3 layers of hippocampus; DG hilus, dentate gyrus hilus; GABA, γ-aminobutyric acid; GCL, granule cell layer; Glut, glutamate; MS/DB, medial septum/diagonal band of Broca; NI, nucleus incertus; PV, parvalbumin; RLN3, relaxin-3; RXFP3, relaxin-family peptide receptor 3; SST, somatostatin; ±, plus or minus (PV).

Septal PV/GABAergic neurons are the main source of GABAergic projections to the hippocampus and specifically target hippocampal interneurons, including somatostatin (SST) and GABA neurons (Freund and Antal, 1988; Freund and Gulyás, 1997), and a number of studies have demonstrated that PV/GABA neuron activity is crucial for hippocampal theta rhythm (Borhegyi et al., 2004; Bassant et al., 2005; Tsanov, 2015), directly modulating cognitive functions, including spatial coding and learning and memory (Vinogradova et al., 1998; McNaughton et al., 2006; Korotkova et al., 2018). For example, in a study to assess the role of hippocampal “rhythmicity” in learning in a MWM test, tetracaine injection into the septum of rats to block theta rhythm by blocking septal inputs to the hippocampus, resulted in an impairment in initial learning in the MWM test, whereas restoring theta-like rhythmicity restored initial learning in the MWM (McNaughton et al., 2006). Thus, on the basis of the behavioral and neuroanatomical findings in the present study, it is proposed that RXFP3-expressing PV/GABA neurons in the MS/DB might regulate learning and long-term memory reference memory, potentially by disrupting hippocampal theta rhythm via septal PV/GABA projections to hippocampal interneurons (Figure 6). Indeed, further studies are now required to examine the effect of septal RXFP3 depletion on theta oscillations as well as studies to examine how other memory processes (i.e., spatial working memory in a spontaneous Y-maze task) are modulated by relaxin-3/RXFP3 signaling within the septal region. In these future studies the potential involvement of RXFP3 effects on septal glutamatergic neurons must also be considered.

Furthermore, the discovery that Rxfp3 mRNA is absent from MS/DB cholinergic neurons in the mouse is consistent with a recent study in the rat (Albert-Gascó et al., 2018), and provides further insights into the direct, and potentially indirect, ways the relaxin-3/RXFP3 system regulates the neural activity in this integrative region. Thus, further comprehensive, quantitative studies of this key neuropeptide signaling network are now warranted, including studies of RXFP3 depletion from specific neuron populations, using viral-based methods and/or relevant Cre driver-reporter mouse lines.

Most recently, the importance of the septum and hippocampal networks in the control initiation of locomotion and the speed of movement has been demonstrated (Fuhrmann et al., 2015), as well as newly identified networks of hippocampal interneurons that are active during movement and immobility (Arriaga and Han, 2017). Indeed, some of these identified SST GABA neurons within hippocampus, may be responsive to relaxin-3, as recent studies in our laboratory have demonstrated close contact between relaxin-3-containing fibers and SST-positive neurons in the mouse DG, CA3 and *stratum oriens* (Haidar et al., 2017), although in studies in which we depleted RXFP3 from the DG only, there was no effect on locomotor activity, despite a significant effect on spatial reference and working memory. Similarly, in the present study, RXFP3 depletion in the MS/DB did not result in locomotor or exploratory deficits, suggesting that the relaxin-3/RXFP3 system does not strongly modulate/control exploratory and locomotor related behaviors via the SHS network in the mouse (Haidar et al., 2017). However, in light of the demonstrated influence of broad *NI* networks on these modalities (Ma et al., 2017), further studies are warranted.

The importance of these septohippocampal networks in different neuropathological conditions has also been identified. For example, hippocampal SST/GABA interneurons were implicated in the deficits in Alzheimer’s disease (AD) in a recent study using a transgenic mouse model of AD (Schmid et al., 2016) and both a loss and abnormal function of SST/GABA interneurons in ventral hippocampus is also implicated in the cognitive symptomology of schizophrenia in studies of animal models of memory deficits (Lodge et al., 2009; Nakazawa et al., 2012). Therefore, the demonstration that a broadly distributed, and functionally diverse population of SST/GABA neurons express RXFP3 in mouse and rat hippocampus (Haidar et al., 2017; Rytova et al., in press), suggests that RXFP3-related signaling may also be disrupted in these neurodegenerative disorders and/or may represent a therapeutic target (Kumar et al., 2017; Ma et al., 2017). Furthermore, given the involvement of the hippocampus and the strong anatomical and functional relationship between the septum and the hippocampus, and behavioral and neuroanatomical findings from the current study, further studies of the MS/DB in neurodegenerative and psychiatric disease models are also warranted (Belarbi et al., 2011; Van der Jeugd et al., 2011; Loreth et al., 2012) and should include investigations of relaxin-3/RXFP3 systems.

## Conclusion

The presence of the relaxin-3/RXFP3 signaling system across the major anatomical nodes of the SHS (Ma et al., 2009b; Smith et al., 2010; Olucha-Bordonau et al., 2012; Haidar et al., 2017; Albert-Gascó et al., 2018), suggests it can regulate those neuronal circuits involved in cognitive processes. This is the first study to investigate the impact of a loss of RXFP3 signaling in the mouse septum on learning and memory, and has revealed novel insights into the role of the relaxin-3/RXFP3 system in spatial learning and long-term reference memory. In addition, our neuroanatomical data complement our behavioral data and provide further evidence of the possible RXFP3-related modulation of septal PV/GABAergic neuron activity and potentially other septal neurons, including a population of glutamatergic neurons, consistent with their ability to modulate SHS physiology and related cognitive processes (Borhegyi et al., 2004; Bassant et al., 2005; Huh et al., 2010; Leão et al., 2015; Tsanov, 2015; Unal et al., 2015; Gangadharan et al., 2016; Robinson et al., 2016; Müller and Remy, 2018).

## Author Contributions

AG conceived and supervised the project. MH and AG designed the experiments. MH, KT, CZ, MN, JC and AW performed the experiments. MH, KT, MN and JR performed the analysis. MH and AG wrote the manuscript. All authors provided editorial comments and approved the final version.

## Conflict of Interest Statement

The authors declare that the research was conducted in the absence of any commercial or financial relationships that could be construed as a potential conflict of interest.
